# Are clinical severity and real-world functioning associated to committing crimes in people with severe mental illness? Results from a cross-sectional study on three cohorts of forensic and non-forensic patients

**DOI:** 10.1192/j.eurpsy.2023.921

**Published:** 2023-07-19

**Authors:** I. Calzavara Pinton, V. Stanga, M. Di Noto, E. Butti Lemmi Gigli, C. Cerati, A. Savorelli, N. Necchini, G. Nibbio, G. Deste, S. Barlati, C. Turrina, A. Vita

**Affiliations:** 1 Department of Clinical and Experimental Sciences, University of Brescia; 2 Department of Mental Health and Addictions, ASST Spedali Civili of Brescia, Brescia, Italy

## Abstract

**Introduction:**

In Italy, subjects with severe mental illness (SMI) considered “in danger of posing a threat to others” are hospitalized into structures known as “*REMS–Residenze per l’Esecuzione delle Misure di Sicurezza*”, designed to provide rehabilitating programs. There are also specialized forensic teams to support Community Mental Health Centers (CMHC) in helping patients who committed crimes. A better characterization of clinical and real-world functioning of forensic patients represents a topic of clinical interest (Caruso R *et al*. Curr Psychiatry Rep 2021; 7 29; Barlati *et al*. Eur Arch Psychiatry Clin Neurosci 2022, *in press*; Fazel *et al*. Br J Psychiatry. 213 609-614).

**Objectives:**

Aims were to compare clinical and psychosocial functioning characteristics in three cohorts of SMI patients.

**Methods:**

A total of 29 patients hospitalized in REMS facilities were included into this study; starting from this first group an equal number of individuals matched for sex, age, and diagnosis were included in other two groups of outpatients cared for by the forensic team and of non-forensic outpatients treated by CMHC. Clinical severity was measured through the Clinical Global Impression scale – Severity (CGI-S) and real-world functioning was measured through the Personal and Social Performance scale (PSP). Analyses included Chi-Square test for categorical variables and Kruskall-Wallis test for continuous variables with Mann-Withney U test for post/hoc comparisons. P values < 0.05 were considered significant.

**Results:**

Significant between-groups differences emerged regarding psychosocial functioning (p=0.013): that was more compromised in the REMS group (mean:34.0) when compared to the forensic team subjects (mean:41.3) and to the subjects in the CMHC group (mean:47.7).

Results concerning clinical severity point in the opposite direction: more severe symptoms were observed in the CMHC group (mean:4.7) compared to the REMS group (mean: 4.3) and the forensic outpatients (mean:3.5). The difference in the CGI-S mean scores is significant for the forensic outpatients when compared to the REMS group (p=0.011) and to the CMHC group (p<0.001).

**Conclusions:**

Specialized teams are central in the managing of forensic patients: of particular interest are the data regarding clinical symptoms severity, which could also be read with a de-stigmatizing focus, highlighting that a worse clinical severity is not associated with being more dangerous to other people and to the society in general.

**Disclosure of Interest:**

None Declared

